# Topology optimization of a heat-assisted magnetic recording write head to reduce transition curvature using a binary optimization algorithm utilizing the adjoint method

**DOI:** 10.1038/s41598-022-18112-z

**Published:** 2022-08-17

**Authors:** Gregor Wautischer, Claas Abert, Florian Bruckner, Florian Slanovc, Dieter Suess

**Affiliations:** 1 Faculty of Physics, University of Vienna, Vienna, Austria; 2 Research Platform MMM Mathematics-Magnetism-Materials, University of Vienna, Vienna, Austria

**Keywords:** Applied physics, Condensed-matter physics

## Abstract

In this work, the possibility to reduce transition curvature in heat-assisted magnetic recording, using a conventional write head design, by shaping the recording field to counteract the circular profile of the heat pulse is investigated. Topology optimization of the head tip is performed in order to create the desired cross-track field profile for increasing distances from the write head tip. For the topology optimization, the adjoint method is utilized to calculate the necessary gradients and a binary optimization scheme is proposed. The optimizations are performed considering linearized material parameters reducing the computational complexity and the results are compared to optimizations incorporating the full non-linear material behavior. The optimized field profiles are evaluated for their influence on the read-back process. To do so, switching probability phase diagrams are calculated and the curvature parameter, the signal to noise ratio and the channel bit density are extracted. The presented results show that while transition curvature can be reduced by shaping the cross-track profile of the write field, this alone does not consequently lead to an improvement of the read back process. Therefore, completely new head designs, considering additional parameters have to be investigated.

## Introduction

Transition curvature represents a significant problem for the read-back process in heat-assisted magnetic recording (HAMR)^1,2^. As shown in^3^ the underlying reason for the curvature is the circular thermal profile inside the magnetic recording media due to the applied heat pulse. Several possibilities to tackle this problem, including split-pole and forked write head designs^4–7^, flattening the thermal profile^7^, as well as using the down-track gradient of the write head^8,9^ have recently been presented. In this work, the possibility to reduce transition curvature using a conventional write head design, by shaping the recording field to counteract the curvature of the heat pulse as proposed in^3,10^ is investigated.

In order to create a cross-track profile of the recording field in such a way that the curvature induced by the thermal profile due to the heat pulse is counteracted, the write head tip is optimized using a topology optimization approach. The optimization is performed for increasing distances from the head tip to account for the fact that the actual write point depends on the distance between write head tip and heat pulse center. The algorithm used is capable of considering the full non-linear B/H curve of magnetic materials. However, this approach is very time consuming. Therefore, the majority of optimizations are performed considering the B/H curve of the magnetic material linearized at the working point to speed up computation. The linearly optimized topologies are then recalculated using a non-linear material law to incorporate saturation effects into the final field profile. For selected distances between write head tip and heat pulse center the optimization is also performed using the full non-linear B/H curve of the magnetic material and the obtained results are compared. The optimized cross-track field profiles are then analyzed for their influence on transition curvature and their ability to improve the read-back process.

## Write head optimization

The write head is optimized using a topology optimization algorithm based on the micromagnetic tool magnum.fe^11^ that uses the finite-element package FEniCS^12^ that was introduced in our previous work^13^. Here, we apply our algorithm for the first time successfully to a real world problem. The algorithm has been extended and is now able to consider non-linear material properties of soft as well as hard magnetic structures defined by a non-linear susceptibility $\chi$ and a remanence magnetization $\varvec{M}_{\text {r}}$. It’s fundamentals are explained in the following.

### Forward problem

To calculate the magnetic stray field, the reduced scalar portential formulation is used. Within this formulation the non-linear magnetostatic forward problem reads

1 $$\begin{aligned} \varvec{\nabla }\cdot \left[ \left( 1+\chi (\varvec{H})\right) \left( \varvec{\nabla }u - \varvec{H}_{\text {ext}}\right) - \varvec{M}_{\text {r}}\right] = 0, \end{aligned}$$

where *u* is the reduced scalar potential, $\varvec{H}_{\text {ext}}$ is the magnetic field produced by the electric coil, $\varvec{M}_{\text {r}}$ is the remanence magnetization and $\chi (\varvec{H})$ is the non-linear suszeptibility. Note that $\chi (\varvec{H})$ indicates the non-linearity of $\chi$ by its dependence on the total magnetic field $\varvec{H}= \varvec{H}_{\text {ext}}+ \varvec{H}_{\text {d}}$, where the induced magnetic field (stray field) $\varvec{H}_{\text {d}}$ is obtained from the scalar potential via $\varvec{H}_{\text {d}}= -\varvec{\nabla }u$. The non-linear forward problem is solved using an inexact Newton method. For the linear systems of equations appearing in each non-linear iteration the open boundary problem is solved using a hybrid FEM-BEM method^14^. This has the advantage that only regions of interest have to be discretized reducing the number of degrees of freedom (dofs).

### Topology optimization

Topology optimization tries to find the optimal topology inside a predefined optimization domain ($\Omega _{\text {opt}}$) without an explicit parameterization^15^. The method used here is the density approach for topology optimization^16^, where a density function

2 $$\begin{aligned} \rho \left( \varvec{x}\right) \in \left[ 0,1\right] \text {, where }\, \rho \left( \varvec{x}\right) = {\left\{ \begin{array}{ll} 0\text {: no material}\\ 1\text {: material} \end{array}\right. } \end{aligned}$$

is introduced inside the optimization region, transforming the remanence magnetization $\varvec{M}_{\text {r}}\rightarrow \rho ^p\varvec{M}_{\text {r}}$ as well as the susceptibility $\chi \rightarrow \rho ^p\chi$. Note, that the parameter *p* is originally introduced to penalize intermediate values of $0 \le \rho \le 1$. However, its greater influence was shown in^13^ where a detailed analysis of the optimization of a soft magnetic flux guide concentrator was performed. The results showed that with increasing *p* the number of dofs with intermediate values of $\rho$ after optimization actually increased, while the performance of the obtained topologies increased in terms of fulfilling the design goal. Therefore, the influence of the penalization parameter *p* was also investigated during the optimizations presented below. The magnetostatic forward problem within $\Omega _{\text {opt}}$ then reads

3 $$\begin{aligned} F\left( u,\rho \right) := \varvec{\nabla }\cdot \left[ \left( 1+\rho ^p\chi (\varvec{H})\right) \left( \varvec{\nabla }u - \varvec{H}_{\text {ext}}\right) - \rho ^p\varvec{M}_{\text {r}}\right] = 0. \end{aligned}$$

The design goal of the optimization finally has to be cast into an objective functional $\hat{J}\left( \rho \right) = J\left( \varvec{H}(\rho ), \rho \right)$ that is to be minimized.

### Adjoint approach

The gradient of the objective functional $\hat{J}\left( \rho \right)$, with respect to the density function $\rho$ that is necessary to perform the optimization can be calculated efficiently utilizing the adjoint approach (see^13^ for a detailed derivation). The adjoint approach allows for the computation of the gradient by solving first the forward problem for a given density distribution $\rho$ to obtain the corresponding magnetic field $\varvec{H}$ and then solving the so called adjoint equation to obtain the adjoint variable $\lambda$. Note that if the forward problem is self adjoint, as is Eq. (3) in its continuous form, the adjoint equation takes the same form as the forward problem. Therefore, the gradient is obtained by solving two similar equation once, while alternatives like the finite difference method in order to calculate the gradient need at least *n* calculations of the forward problem, where *n* is the number of dofs within the optimization model. After obtaining the adjoint variable $\lambda$ the gradient is given by

4 $$\begin{aligned} \varvec{\nabla }_{\rho }\hat{J} = \left( p\rho ^{p-1}\left[ \chi (\varvec{H})\left( \varvec{\nabla }u-\varvec{H}_{\text {ext}}\right) - \varvec{M}_{\text {r}}\right] \right) \cdot \varvec{\nabla }\lambda + \mathcal {R}\left( \frac{\partial J}{\partial \rho }\right) , \end{aligned}$$

where $\mathcal {R}$ is the corresponding Riesz representer. Note, that the non-linearity of the forward problem does not influence the adjoint approach. This is since the adjoint equation is a linear differential equation. The total magnetic field $\varvec{H}$, on which the susceptibility $\chi \left( \varvec{H}\right)$ in Eq. (4) depends, is the total magnetic field of the current topology, obtained by solving the forward problem.

### Binary optimization algorithm

**Figure 1:**
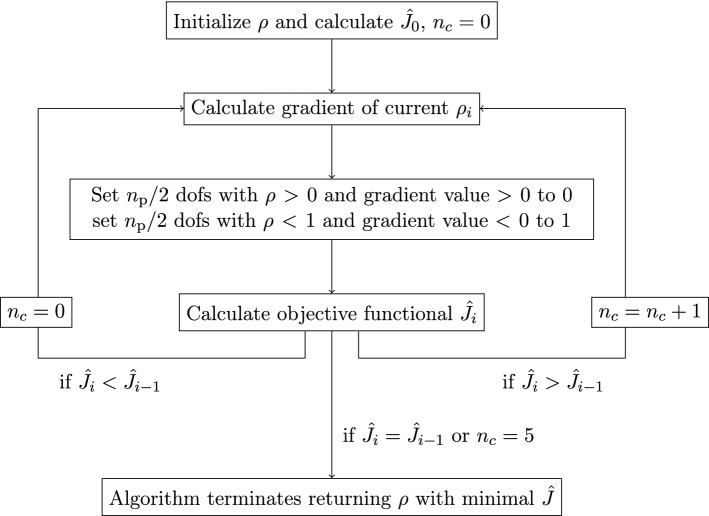
Flowchart of the binary topology optimization (BTO) algorithm.

Using the gradient obtained from the adjoint method a topology optimization problem can be solved using a gradient based optimization algorithm. Since the goal is to obtain a topology with the density function $\rho$ being 0 or 1 without intermediate values, using a continuous optimization algorithm like steepest decent, conjugate gradient or similar, with or without line search makes the usage of some kind of regularization necessary. This can be achieved by using an additional regularization term (Tikhonov regularization) or by brute force regularization where all dofs with $\rho \le 0.5$ are set to 1 and all dofs with $\rho < 0.5$ are set to 0 after the optimization terminates^13^. In order to avoid such a procedure a binary optimization algorithm can be used. The idea here is, that only values of $\rho = 0$ or 1 are physically meaningful and hence intermediate values have to be suppressed as e.g. exploited in^17–19^. Here, a local, gradient based, binary optimization algorithm is implemented. The algorithm is visualized in Fig. 1. It accepts a parameter $\eta \in (0,1]$ that defines the fraction of dofs participating in each optimization step as $n_{\text {p}} = \eta \cdot n_{\text {dofs}}$ where $n_{\text {dofs}}$ is the number of degrees of freedom in the optimization region $\Omega _{\text {opt}}$. Given an initial scalar indicator function $\rho$, an iteration starts by first calculating the gradient. In a next step the gradient is analyzed and the $n_{\text {p}}/2$ dofs for which the gradient is largest positive (removing material is most advantageous) and their value of $\rho$ is unequal 0, are set to 0. Then, the $n_{\text {p}}/2$ dofs for which the gradient is larges negative (adding material is most advantageous) and their value of $\rho$ is unequal 1 are set to 1. Thereafter, the objective functional is evaluated using the updated $\rho$. If the updated $\rho$ reduces the objective functional value it is accepted and the next iteration starts again by calculating the gradient. If the objective functional stays constant i.e. no dofs of $\rho$ have changed during the last iteration, a minimum is found and the algorithm terminates. If the objective functional value increases, up to five further iterations ($n_c \le 5$) are performed to avoid a premature termination in a local minimum. If no reduction of the objective functional value is found within these five iterations, the algorithm terminates as well, returning the topology with the lowest objective functional value. Note that the algorithm is independent of the overall size of the gradient, but only the relative size of the gradient at each dof is of importance.

### Write head design parameters

**Figure 2:**
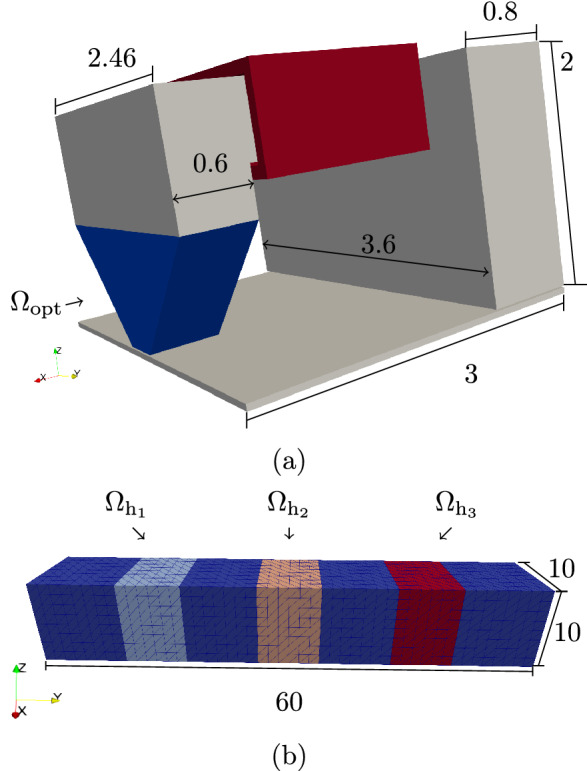
(**a**) Model of the write head (optimization region $\Omega _{\text {opt}}$ highlighted in blue and coil highlighted in red; dimensions in µm) and (**b**) mesh of the fieldbox used for the topology optimization procedure (dimensions in nm).

The model used for the optimizations can be seen in Fig. 2. The basic dimensions have been taken from^5^ with only the head tip altered to be used as optimization domain $\Omega _{\text {opt}}$. $\Omega _{\text {opt}}$ has a height (*z*-direction) of 1 µm and at the bottom has a width (*y*-direction) of 400 nm. The model includes an underlayer with a thickness of 50 nm at a distance of 15 nm from the recording head. The fieldbox within which the magnetic field is evaluated is shown in Fig. 2b. Its vertical distance from the head as well as from the underlayer is 2.5 nm. The mesh used consists in total of 130,029 nodes (678,511 elements) where 103,616 nodes (582,328 elements) belong to the optimization region and 3969 nodes (18,432 elements) belong to the fieldbox. Note that in order to increase the topology’s resolution where it is most important, the optimization region is divided into three areas with different mesh resolution, where the area next to the fieldbox, at the inner edge of the optimization region is meshed the finest.

The optimization was performed for down-tack distances of the fieldbox center of $d = 0, 10, 20, 30, 40$ and 50 nm from the inner edge of the write head tip (negative *x*-direction).

The material of the write head was chosen according to^20^ to have a saturation flux density of $B_{\text {s}} = 2.4$ T and an initial susceptibility of $\chi _{\text {0}} = 1200$. For the topology optimization using a linear material law, $\chi$ was taken as equal to $\chi _0$ giving

5 $$\begin{aligned} B\left( H\right) = \mu _0 \left( 1+\chi _0\right) H .\end{aligned}$$

For the non-linear optimization, the material was modeled as an isotropic non-linear material represented by a hyperbolic tangent

6 $$\begin{aligned} B\left( H\right) = \mu _0 \left( M_{\text {s}}\cdot \tanh \left( \chi _{\text {0}}/M_{\text {s}}\cdot H\right) +H\right) , \end{aligned}$$

where $$M_{\text {s}} = B_{\text {s}}/\mu _0$$ and $$H = \left| \varvec{H}\right|$$.

**Figure 3:**
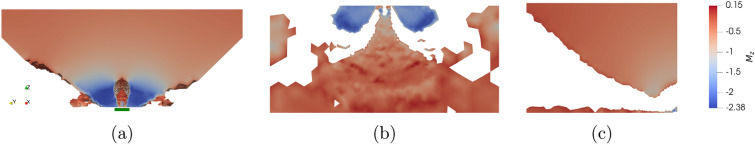
(**a**) Front view (including fieldbox in green), (**b**) bottom view slice and (**c**) side view slice through the middle of the linearly optimized write head tip topology for a fieldbox distance of 10 nm.

Since the coil dimensions were chosen arbitrarily the current density was adjusted so the write head tip is nearly saturated (see Fig. 3) and was kept constant for all presented non-linear calculations. Note however, that for the linear optimization the exact value of the current density is insignificant due to the linear material law.

For the optimization the initial value of the density function $\rho$ was set to $\rho _{\text {init}} \equiv 0.5$. The density function $\rho$ as well as $\chi$, $\varvec{M}_{\text {r}}$, $\varvec{H}_{\text {ext}}$ and the derived stray field $\varvec{H}_{\text {d}}= -\varvec{\nabla }u$ and $\varvec{\nabla }\lambda$ are constant within each element while the potential *u* and the adjoint variable $\lambda$ are calculated using piecewise linear basis functions $\left( \mathcal {P}_1\right)$.

The curvature of the heat pulse used for HAMR creates a heat profile in cross-track (*y*-)direction in such a way that the material is heated less away from the track center^3^. Since at lower temperatures a higher write field is necessary in order to write the magnetic bits, the write field generated by the write head has to be shaped to counteract this. The *z*-component (the field component responsible for writing the perpendicular bits) of the write field, therefore has to be maximized away from the tack center where temperature is lowest and minimized at the track center where temperature is highest. This is achieved by introducing three equally large volumes inside the fieldbox, two on each side ($\Omega _{\text {h}_1}$ and $\Omega _{\text {h}_3}$) and one in the middle (($\Omega _{\text {h}_2}$) as also depicted in Fig. 2b. By minimizing the objective functional:

7 $$\begin{aligned} \hat{J} = \frac{1}{2}\int _{\Omega _{\text {h}_1}} H_{z}\,\text {d}V-\int _{\Omega _{\text {h}_2}} H_{z}\,\text {d}V+\frac{1}{2}\int _{\Omega _{\text {h}_3}} H_{z}\,\text {d}V, \end{aligned}$$

the difference of the *z*-field component between the center evaluation area ($\Omega _{\text {h}_2}$) and the two evaluation areas on the sides of the fieldbox ($\Omega _{\text {h}_1}$ and $\Omega _{\text {h}_3}$) is then maximized, leading to the desired field shape. The three evaluation areas all have a cross-track (*y*-) length of 8 nm and $\Omega _{\text {h}_1}$ and $\Omega _{\text {h}_3}$ are centered around $y = \pm 16$ nm.

### Results

#### Linear optimization

**Figure 4:**
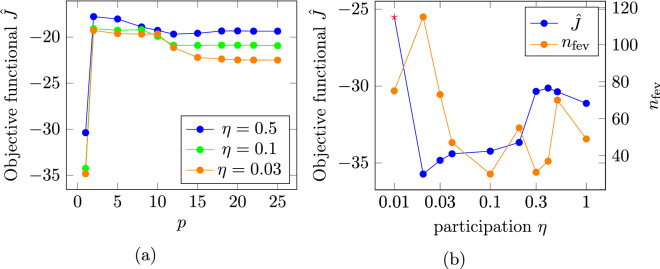
(**a**) Dependence of the objective functional value $\hat{J}$ on the parameter *p* for different participation values $\eta$ and (**b**) dependence of the objective functional value $\hat{J}$ and number of necessary function evaluations $n_{\text {fev}}$ on the participation parameter $\eta$, both for the linear topology optimization at a distance of $d = 10$ nm. The red stars indicate simulations for which the optimization terminates prematurely.

For the linear optimization, as shown in Fig. 4a for a fieldbox distance of $d = 10$ nm the best topology in terms of having the lowest objective functional value is found for $p=1$ for different values of $\eta$. This is also true for other values of *d* and is in contrast to the findings published in^13^ where for high susceptibility materials larger values of *p* were found to be beneficial. Regarding the objective functional value’s dependence on the participation parameter $\eta$, Fig. 4b shows a decreasing objective functional value with decreasing $\eta$, where $\rho$ is set to 0 or 1 for all dofs, until for $\eta = 0.01$ the optimization terminates prematurely with $\rho$ still having the initial value for some dofs and a higher objective functional value. At $\eta = 0.1$ the number of necessary function evaluations $n_{\text {fev}}$ has a minimum of 30. This behavior is similar for all distances in so far, as for $\eta > 0.04$ all optimizations find an optimal topology with $\rho$ set to 0/1 for all dofs. Furthermore, for all distances, the smoothness of the resulting topologies decreases for $\eta < 0.1$ and they become increasingly irregular while the general topology stays unchanged.

For the presented evaluations, therefore, the solutions for $\eta = 0.1$ are taken. In Fig. 3 the optimized topology for a distance of 10 nm, $p=1$ and $\eta = 0.1$ is shown. The optimized write head has a slit similar to the split pole geometries presented in^4–7^. However, additionally between the two prongs material is present. This material extends as an island behind the write head tip (see bottom view in 3b and side view in Fig. 3c) and increases the field difference between the center and the sides of the fieldbox, since it channels magnetic flux away from the fieldbox center.

To incorporate saturation effects into the evaluation, the magnetic fields produced by the linearly optimized topologies were recalculated using the isotropic non-linear material law as introduced in Eq. (6).

**Figure 5:**
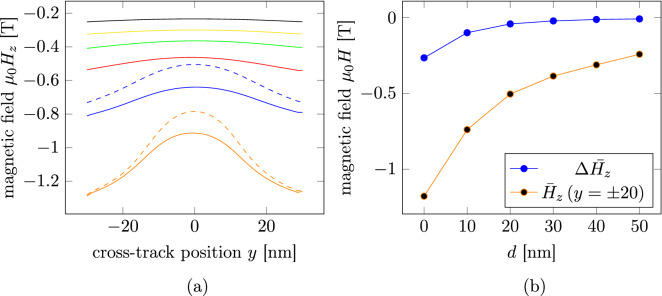
(**a**) Cross-track field profiles at different distances *d* from the write head tip for the linearly optimizations (continuous lines) and the non-linear optimizations (dashed lines) obtained from the respective optimized topologies and (**b**) dependence of the field difference $\Delta \bar{H_{z}}$ and the mean maximal field $\bar{H}_{z}\left( y=\pm 20\right)$ on the down-track distance from the head tip *d* for the linear optimizations.

In Fig. 5a the cross-track field profiles evaluated along the center of the fieldboxes are plotted. It is clearly visible that with increasing distance the maximum field strength as well as the field difference between the center and the sides decreases rapidly. This is also shown in Fig. 5b where the mean field $\bar{H}_{z}\left( y=\pm 20\right) = \frac{H_{z}\left( 20\right) +H_{z}\left( -20\right) }{2}$ and the field difference $\Delta \bar{H_{z}} = H_{z}\left( 0\right) - \bar{H}_{z}\left( y=\pm 20\right)$, where $H_{z}\left( y\right)$ is the *z*-component of the magnetic field at cross-track position *y*, is plotted versus the distance of the field profiles. The field profile at $d = 0$ nm distance from the write head tip shows a field difference of ${\mu _0} \Delta \bar{H_{z}} = 0.27$ T.

#### Non-linear optimization

**Figure 6:**
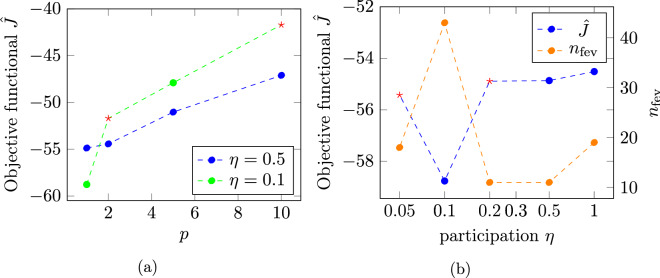
(**a**) Dependence of the objective functional value $\hat{J}$ on the parameter *p* for different participation values $\eta$ and (**b**) dependence of the objective functional value $\hat{J}$ and number of necessary function evaluations $n_{\text {fev}}$ on the participation parameter $\eta$, both for the non-linear topology optimization at a distance of $d = 10$ nm. The red stars indicate simulations for which the optimization terminates prematurely.

**Figure 7:**
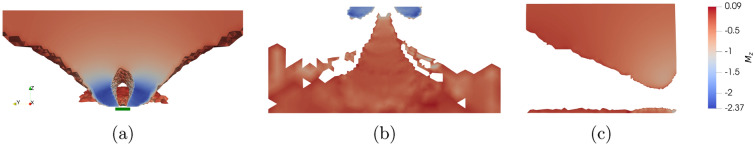
(**a**) Front view (including fieldbox in green), (**b**) bottom view slice and (**c**) side view slice through the middle of the non-linearly optimized write head tip topology for a fieldbox distance of 10 nm.

In order to investigate the influences of saturation effects during optimization, for a distance of $d = 0$ and 10 nm, the optimization was repeated, using a non-linear material law (Eq. (6)). Similar to the linear optimization, for all distances *d*, the best topology can be found for $p=1$ for different values of $\eta$ as shown in Fig. 6a for $d = 10$ nm. In Fig. 6b the dependence of the objective functional value and of the number of necessary function evaluations on the participation $\eta$ for $d = 10$ nm is shown. The best topology is found for $\eta = 0.1$ while for $\eta = 0.2$ and 0.05 the optimization terminates prematurely with some dofs still having initial values of $\rho$. The optimized topology is shown in Fig. 7. Compared to the topology obtained with the linear optimization, the main features are also present, but changed in size and position. The optimized field profile (see dashed blue line in Fig. 5a) has a field difference of ${\mu _0}\Delta \bar{H_{z}} = 0.14$ T, an increase of 42.38 % with respect to the linearly optimized field profile. The optimization with $d = 0$ nm terminates prematurely also with $\eta = 0.1$. The field profile of the optimization with $\eta = 0.5$ (see dashed orange line in Fig. 5a) shows a field difference of ${\mu _0}\Delta \bar{H_{z}} = 0.36$ T. This constitutes an increase of 39.07 %.

Regarding computational effort, while for $d = 10$ nm for the linear optimization with $\eta = 0.1$, solving the forward problem on average needs 4 linear iterations, using the non-linear material law this increases to on average 357 linear iterations necessary to solve the forward problem using the inexact Newton method. For $d = 0$ the number of linear iterations increases from also an average 4 for the linear optimization with $\eta = 0.1$ to on average of 252 for the non-linear optimization with $\eta = 0.5$. Note that solving the adjoint equation requires the same computational effort since it is a linear equation in both cases.

In conclusion, the topologies optimized using a linearly approximated material law produce the same main features as the non-linearly optimized topologies. However, the features, namely the size of the two prongs, the size of the gap between the prongs and the size and position of the material island between the prongs are different. Furthermore, using a non-linear material law increases the desired field difference in cross-track direction by about 40 %. This shows, the necessity of incorporating saturation effects by using the full non-linear material characteristics. However, this also increases the computational effort of the forward field calculation by about two magnitudes. Furthermore, in comparison to the field difference of 1.05 T reported as necessary to completely suppress transition curvature by^3^, the maximum field difference of 0.36 T found is much lower. While the mean field strength of $\mu _0 \bar{H}_{\text {z}}\left( y=\pm 20\right) = 1.15$ T is around the proposed value of 1.4 T, the field strength at the center of 0.78 T is much higher then the reported necessary value of 0.35 T.

## Curvature reduction

**Table 1:** Material parameters and dimensions of the FePt grains.

Curie temp.$T_C$(K)	Damping$\alpha$	Anisotropy const. $K_1$(MJ/m$^3$)	Exchange$A_{\text {ex}}$(pJ/m)	Saturation polarization$J_s$(T)	Height h (nm)	Diameter d (nm)
693.53	0.02	6.6	28	1.35	8	5

**Figure 8:**
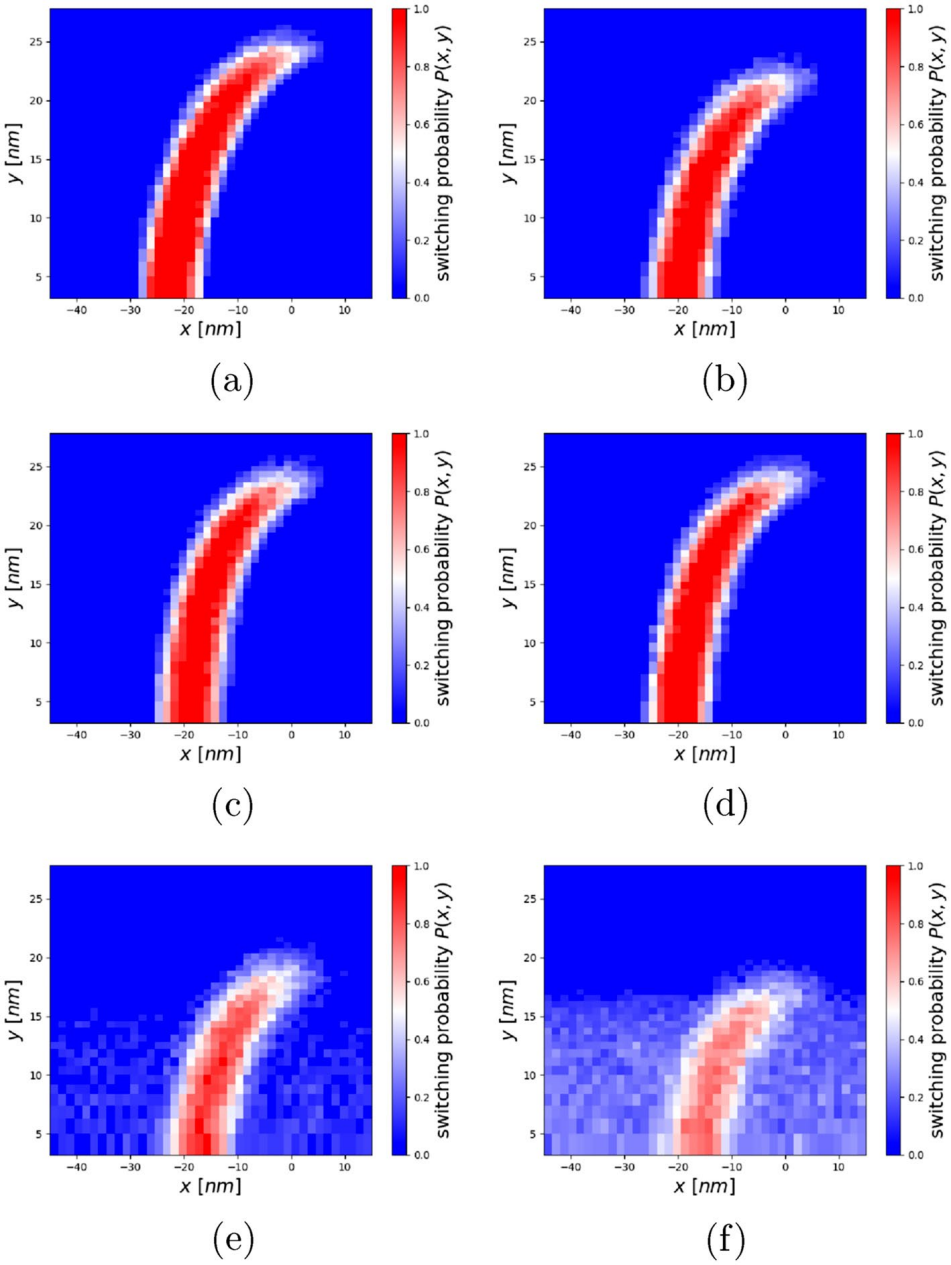
Switching probability phase diagrams for the constant fields with (**a**) 1.3 T and (**b**) 0.9 T, the non-linear optimized field profile at (**c**) $d = 0$ nm and the linear optimized field profiles at (**d**) $d = 0$ nm, (**e**) $d =$ 20 nm and (**f**) $d =$ 40 nm.

In order to evaluate the optimized field profiles, their influence on the read-back process is investigated. To do so, first switching probability phase diagrams (SPPD)^21^ are calculated for the optimized field profiles as well as for homogeneous field profiles with a constant strength of 0.9, 1.1 and 1.3 T. The SPPD shows the switching probability of a recording grain as a function of its down-track *x* and cross-track position *y* on the writing track. For the calculation, an FePt-like hard magnetic granular recording medium with material parameters as presented in Table 1 is used. The heat profile is modeled as Gaussian with a full width at half maximum (FWHM) of 60 nm as

8 $$\begin{aligned} T\left( x, y, t\right) =\left( T_{\text {max}} - T_{\text {min}}\right) e^{\frac{\left( x-vt\right) ^2+y^2}{2\sigma ^2}} + T_{\text {min}} \end{aligned}$$

where, $\sigma = \frac{FWHM}{\sqrt{8\ln \left( 2\right) }}$, $T_{\text {max}}=753$ K is the maximum temperature, $T_{\text {min}}= 300$ K is the temperature of the recording medium without the heat pulse and a velocity of the write head of $v=15$ m/s is considered. The applied field is tilted by 22$^\circ$ with respect to the normal direction and is modeled as trapezoidal with a field duration of 0.57 ns and a switching time of 0.1 ns per bit, which results in a bit length of 10 nm. Each point of a phase diagram contains 100 simulated switching trajectories.

In Fig. 8 the SPPDs of the linearly and non-linearly optimized field profiles at different distances from the write head tip, as well as for homogeneous fields with a strength of 0.9, and 1.3 T are shown. It can be seen that for a lower field strength at the center, the switching probability is shifted in positive down-track position, representing higher writing-temperature values and the C-shape becomes deformed. Furthermore, the possibility to switch grains with a large cross-track distance is reduced for lower field values at these positions. Note, that for $d > 10$ nm the phase diagrams for the optimized field profiles show a non-zero switching probability for a wide range of down-track positions since the field becomes to weak for a reliable writing process (Fig. 8e,f).

From the phase diagrams, the curvature parameter *c*, the signal to noise ratio (SNR) and the channel bit density (CBD) are extracted. The curvature parameter, defined as $c = \frac{\Delta x}{\Delta y}$, where $\Delta x$ is the down-track range and $\Delta y$ is the cross-track range in which the bit is written with a probability $P \ge 50 \%$^9^ can directly be taken from the phase diagrams. The signal to noise ratio (SNR) constitutes a quality criterion for a written bit track. To calculate the SNR, the read-back signal $V\left( x\right)$ is considered as a random variable with expectation value $\mathbb {E}\left[ V\left( x\right) ^2\right]$ and variance $\mathbb {V}\left[ V\left( x\right) \right]$^22^. Using the signal power SP$= \int _{x_{\text {start}}}^{x_{\text {end}}} \mathbb {E}\left[ V\left( x\right) ^2\right] \;\text {d}x$ and the noise power NP$= \int _{x_{\text {start}}}^{x_{\text {end}}} \mathbb {V}\left[ V\left( x\right) \right] \;\text {d}x$ of a bit pattern between down-track positions $x_{\text {start}}$ and $x_{\text {end}}$ the SNR is defined as

9 $$\begin{aligned} \text {SNR} = \frac{\rm {SP}}{\rm {NP}}. \end{aligned}$$

Note, that here a probability mapping approach according to^23^ is used to obtain the expectation value and the variance. Furthermore, the CBD^24^ is defined as the pulse width at the $50\%$ amplitude point of the differentiated reader response to an isolated transition divided by the bit length and is therefore a measure for the sharpness of the transition between two consecutive opposing bits.

**Table 2:** SNR, curvature *c* and CBD values for different cross-track field profiles.

Field profile	SNR (dB)	Curvature *c*	CBD
Not optimized constant 0.9 T	16.48	1.05	1.54
Not optimized constant 1.1 T	17.47	1.07	1.51
Not optimized constant 1.3 T	17.90	1.10	1.50
Non-linear optimized $d = 0$ nm	16.19	0.92	1.48
Linear optimized $d = 0$ nm	17.26	0.95	1.48
Non-linear optimized $d = 10$ nm	10.49	0.83	1.57
Linear $d = 10$ nm	13.28	1.04	1.57
Linear optimized $d = 20$ nm	8.91	0.86	1.60
Non-linear optimized, shifted $d = 0$ nm	17.17	0.92	1.46

**Figure 9:**
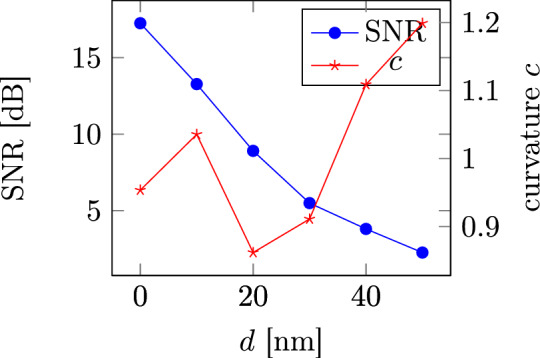
SNR and curvature *c* values for the optimized field profiles in dependence of their distance from the write head tip *d*.

**Figure 10:**
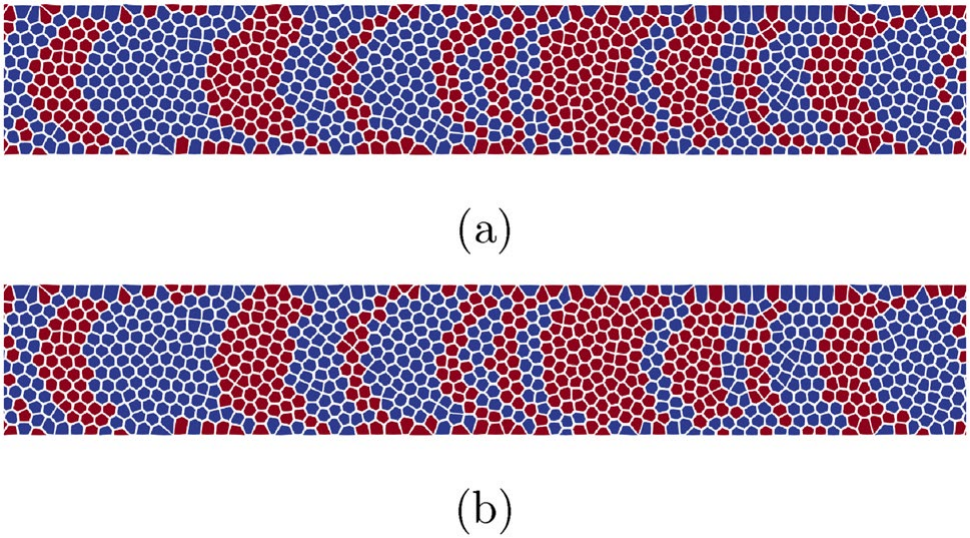
Bit series written on the basis of the phase diagrams for (**a**) a field of 1.3 T constant in cross-track direction and (**b**) the shifted, non-linear optimized field profile at a distance of $d =$ 0 nm from the write head tip.

The SNR, CBD and *c* values are shown in Table 2 for constant field profiles with 0.9, 1.1 and 1.3 T as well as for the optimized field profiles with a distance of 0, 10 and 20 nm from the write head. It can be seen that in comparison to the constant field profiles, the optimized field profiles show a reduced curvature *c* as was the goal of the topology optimization. Furthermore, the non-linearly optimized field profiles, having a higher field difference $\Delta \bar{H_{z}}$ compared to the linearly optimized field profiles also have a lower curvature *c*. In Fig. 9 the curvature *c* and SNR values are plotted in dependence of the distance *d* for the linearly optimized field profiles. It can be seen, that the dependence of the curvature *c* on the distance from the write head tip is not straight forward. This is due to the dependence of the curvature parameter on the bit length in cross-track direction $\Delta x$ that changes drastically with increasing *d*. It is furthermore visible, that a lower curvature *c* does not cause a higher SNR value. This is due to the fact that the SNR value mostly depends on the write field strength at the center of the bit ($y = 0$). To illustrate this, the non-linearly optimized field profile at $d = 0$ nm is shifted by 0.16 T to have a field strength of 0.9 T at the bit center. The resulting profile then shows a similarly low curvature *c* and an even lower CBD while having a larger SNR value (see last entry in Table 2). It can therefore be concluded that transition curvature can be reduced and the read back process can be improved by shaping the topology of the write head tip. However, this can only be done by increasing the write field in cross-track direction while maintaining an as large as possible write field at the bit center to not lose SNR. Finally, in Fig. 10 two bit series with a cross-track width of 60 nm, written on the basis of the phase diagrams are shown. A curvature reduction for the optimized field profile can be seen.

## Conclusion

Using a binary topology optimization algorithm the cross-track field profile of a write head was optimized to reduce transition curvature as suggested in^3,10^. In detail, the vertical field component responsible for writing the perpendicular bits, was optimized to be minimal at the center and to increase in cross-track direction. The optimization was performed for different distances from the write head tip to account for the fact that the point at which a bit is written depends on an interplay of the write mediums temperature and the applied field strength and therefore also on the distance between heat pulse and write head. The optimizations were performed considering the full non-linear material characteristics as well as using a linear approximation reducing computational complexity tremendously. While the linearly optimized topologies show the same main features as non-linearly optimized topologies, an increase in field difference of about 40 % can be gained by using non-linear optimization. The optimizations delivered a new kind of split-pole like topologies with additional material between the two poles. The maximal generated field difference between the center and the sides at 20 nm distance in cross-track direction was 0.36 T for a position directly at the write head tip’s edge. For larger distance from the write head, the field difference as well as the maximum field decrease quickly.

The optimized field profiles were evaluated for their influence on the read-back process. A reduction in transition curvature was achieved for the optimized field profiles with a distance below 40 nm from the write head tip edge with respect to fields constant in cross-track direction. The non-linearly optimized field profile directly at the head’s tip edge showed the reduction in curvature of 16.4 % with respect to the curvature of a constant field in cross-track direction of 1.3 T. Generally, the non-linearly optimized field profiles, showing a larger cross-track field difference also produce a lower transition curvature. It is therefore concluded, that by optimizing the topology of the write head tip the transition curvature can be improved, but not fully eliminated. However, the reduction in curvature does not automatically translate into an increase in signal to noise ratio (SNR). This is, since the SNR mainly depends on the field strength at the bit center. Our results therefore suggest, that trying to improve the read back process in heat-assisted magnetic recording by solely reducing transition curvature via shaping the cross-track field profile is not sufficient. It is rather necessary to also consider additional parameters like the field strength at the bit center, making it necessary to investigate completely new head designs to further optimize the write process for heat assisted magnetic recording.

## Data Availability

The data generated and analysed during the current study are available from the corresponding author on reasonable request.
